# Botulinumtoxin Type-A (BoNTA) in the Management of Refractory Trigeminal Neuralgia: An Expert-Opinion, Practice-Oriented Narrative Review on Behalf of the GRASP Study Group

**DOI:** 10.3390/toxins18060248

**Published:** 2026-05-29

**Authors:** Andreas A. Argyriou, Emmanouil V. Dermitzakis, Dimitrios Rikos, Georgia Xiromerisiou, Panagiotis Soldatos, Maria Chondrogianni, Eleni Mavraki, Michail Vikelis

**Affiliations:** 1 Headache Outpatients Clinic, Agios, Andreas Hospital, 26335 Patras, Greece; 2 General Clinic Thessaloniki, 54645 Thessaloniki, Greece; manolis.dermitzakis@gmail.com; 3 404 General Military Hospital, 41222 Larissa, Greece; rikosd@hotmail.com; 4 Neurology Institute, Mindful Mind, 55133 Thessaloniki, Greece; georgiaxiromerisiou@gmail.com; 5 Independent Researcher, 24100 Kalamata, Greece; soldatosp@gmail.com; 6 Second Department of Neurology, National and Kapodistrian University of Athens, School of Medicine, “Attikon” University Hospital, 12462 Athens, Greece; mariachondrogianni@hotmail.gr; 7 Department of Neurology, University General Hospital of Alexandroupolis, 68100 Alexandroupolis, Greece; emavr2009@hotmail.com; 8 Glyfada Headache Clinic, 16675 Athens, Greece; mvikelis@headaches.gr

**Keywords:** refractory, trigeminal neuralgia, pharmacotherapy, botulinumtoxin type-A, BoNTA

## Abstract

Trigeminal neuralgia (TN) ranks among the most excruciating neuropathic pain syndromes, characterized clinically by multiple daily episodes of unilateral, paroxysmal, electric shock-like facial pain. The daily activities and quality of life of affected patients are profoundly diminished. First-line pharmacological agents, such as carbamazepine and oxcarbazepine, provide initial relief for many patients. However, a significant proportion eventually develops refractory symptoms or experience intolerable adverse effects, leading to the discontinuation of traditional oral medications. For these patients with complex clinical phenotypes who fail to respond or are intolerant to these therapies, alternative pharmacological strategies are required before considering invasive surgical procedures. Over the past two decades, botulinumtoxin type-A (BoNTA) has become an effective and safe, minimally invasive therapeutic option for refractory TN. This review provides a practical framework for BoNTA use in the clinical setting of refractory TN. To connect the pathophysiological background with clinical patient care, we summarize the current understanding of TN pathophysiology, the proposed mechanisms by which BoNTA exerts its antinociceptive effects and the evolving clinical evidence supporting its efficacy and safety. We also critically examine dosing protocols, injection techniques, long-term outcomes and the integration of BoNTA into the management algorithm of refractory TN.

## 1. Introduction

Trigeminal neuralgia (TN) is a debilitating chronic neuropathic pain condition that profoundly diminishes the quality of life of affected patients. According to the International Classification of Headache Disorders, 3rd edition (ICHD-3), TN is defined as a painful neurological disorder characterized by recurrent, unilateral, electric shock-like lancinating attacks. These paroxysms are abrupt in onset and termination, strictly limited to the distribution of one or more branches of the trigeminal nerve [1]. According to estimates, the global, pooled incidence of TN stands at approximately 25.3 cases per 100,000 person-years, with an annual prevalence of 45.4 cases per 100,000 inhabitants. TN predominantly affects middle-aged and older individuals, between the ages of 50 and 60, with a higher prevalence in woman [2]. The right side of the face is reported to be slightly more often affected than the left, though bilateral involvement can rarely occur [3].

Typical TN attacks are brief, lasting from few seconds up to two minutes. However, clinical experience and emerging data indicate that some patients experience a constant background pain between typical TN paroxysms [4]. The neuralgiform facial pain experienced during attacks is often triggered by innocuous mechanical stimuli, such as light touch, talking, drinking or eating, while the trigger zones are typically located in the perioral or nasal regions. Patients frequently refuse to eat, speak or wash their face to avoid stimulating these zones and eliciting pain. Such major lifestyle changes, combined with the unpredictable and severe phenotype of TN paroxysms, drives patient’s life in profound psychological distress, manifested with anxiety, depression, sleep disturbances and social withdrawal [5,6].

The initial pharmacological approach to TN relies on sodium channel blockers, such as carbamazepine and oxcarbazepine, which serve as the cornerstone of first-line therapy. Although they provide substantial pain relief for the majority of patients during the early stages of TN, their efficacy often diminishes over time due to a wear-off phenomenon [7]. Consequently, dose escalation becomes inevitable, often accompanied by dose-limiting adverse events such as hyponatremia, dizziness, cognitive impairment and hepatotoxicity. Due to the severity of such toxicities, many patients, especially elderly ones who comprise the majority of the TN population, become intolerant to these adverse events and discontinue treatment early [8].

When patients fail to respond to adequate dosages of at least two traditional oral medications, or when side effects necessitate discontinuation, the condition is termed as a refractory TN [9]. For patients with refractory TN, invasive surgical procedures are to be considered. Microvascular decompression (MVD) associated with neurovascular compression is the ideal next step offering high rates of long-term, medication-free analgesia [10,11]. Neuroablative procedures, such as radiofrequency thermocoagulation, glycerol rhizotomy and stereotactic radiosurgery, are alternative options, particularly for elderly patients or those with significant comorbidities [12,13]. Nonetheless, these invasive procedures carry an increased risk of complications associated with posterior fossa surgery or ablation techniques, including facial numbness, dysesthesia, anesthesia dolorosa, and headache secondary to cerebrospinal fluid leak [14].

In this challenging therapeutic landscape, botulinumtoxin type-A (BoNTA) has become a safe, effective and minimally invasive alternative pharmacotherapy. BoNTA was originally used to manage dystonia and spasticity because of its potent muscle-relaxing properties. We now recognize that it also exerts profound neuromodulatory effects, capable of altering both peripheral and central pain pathways [15].

Several systematic reviews and meta-analyses have summarized the efficacy and safety of BoNTA in TN. The present narrative review is therefore not intended to duplicate those quantitative syntheses, but to provide a practical framework with incremental value for BoNTA use in the clinical setting of refractory TN. To connect the pathophysiological background with practical patient care, we summarize the current understanding of TN pathophysiology, the proposed mechanisms by which BoNTA exerts its antinociceptive effects and the evolving clinical evidence supporting its efficacy and safety. We also critically examine dosing protocols, injection techniques, long-term outcomes, safety monitoring, and the integration of BoNTA into the management algorithm of refractory TN.

For this narrative review, we searched PubMed/MEDLINE, Scopus, Web of Science, the Cochrane Library, and ClinicalTrials.gov for English-language studies and reviews evaluating BoNTA in TN and orofacial neuropathic pain from 2010 until now. Searches combined the terms “trigeminal neuralgia”, “refractory trigeminal neuralgia”, “botulinum toxin”, “botulinum toxin type A”, “BoNTA”, “BTX-A”, “onabotulinumtoxinA”, “lanbotulinumtoxinA”, “injection technique”, “orofacial pain” and “neuropathic pain”. We prioritized randomized controlled trials, systematic reviews, meta-analyses, prospective or retrospective clinical studies, case series, and clinically relevant mechanistic studies. In addition, reference lists of eligible publications were screened to identify additional relevant studies. Because this was a narrative review, no formal risk-of-bias scoring or quantitative meta-analysis was performed; however, the level and limitations of the available evidence are discussed.

## 2. Pathophysiology of TN and the Rationale for Neuromodulation

The pathophysiology underlying TN is complex and multifactorial. The disorder is generally classified into three categories based on its etiology: (i) classical TN, caused by morphological changes in the trigeminal nerve root due to neurovascular compression, typically by the superior cerebellar artery at the root entry zone; (ii) secondary TN, attributed to an identifiable underlying neurological disease, such as a space-occupying lesion or multiple sclerosis affecting the trigeminal pathways; and (iii) idiopathic TN, which has no apparent structural cause [1].

Regardless of the specific etiologic classification, the cornerstone pathophysiological mechanism involves focal demyelination of the primary trigeminal afferents, disrupting the normal signal transport through the nerve fibers, to eventually generate a state of profound hyperexcitability [16]. The affected neuraxons become particularly susceptible to ectopic impulse generation and ephaptic transmission. Consequently, innocuous tactile stimuli carried by large-diameter A-beta fibers inappropriately activate nociceptive A-delta and C fibers. This gives rise to allodynia, transforming a light touch into a perception of agonizing pain [17].

Allodynia, the clinical hallmark of central sensitization, arises from chronic peripheral nerve injury combined with sustained ectopic firing, both of which trigger a cascade of changes within the central nervous system. These neuroplastic changes involve the upregulation of voltage-gated sodium channels, particularly the NaV1.3, NaV1.7 and NaV1.8 subtypes, within the trigeminal ganglia and nucleus caudalis [18,19]. Furthermore, there is an enhanced release of excitatory neurotransmitters and neuropeptides within the trigeminal nucleus caudalis, including glutamate, substance P, and calcitonin gene-related peptide (CGRP) [20]. Functional magnetic resonance imaging (fMRI) studies indicate that these peripheral and central dysfunctions amplify nociceptive signaling into a chronic pain state, making it highly resistant to conventional pharmacotherapies [21,22,23].

Notably, Devor and colleagues proposed the “ignition hypothesis”, based on the interplay between peripheral nerve damage and central neuroplasticity [24]. According to this theory, demyelinated trigeminal roots generate ectopic spontaneous action potentials toward the central nervous system. This not only triggers TN attacks but also exerts long-lasting changes in the excitability of second-order neurons in the brainstem, rendering them hyper-responsive to both noxious and innocuous stimuli. The sustained hyperexcitability of central pathways is further augmented by the release of pro-inflammatory cytokines and neurotrophic factors from activated microglia and astrocytes within the trigeminal nucleus [24].

Therefore, neuromodulation to manage refractory TN by disrupting this pathogenetic interplay between peripheral ectopic firing and central sensitization represents a rational approach. Conventional pharmacological agents primarily aim to inhibit axonal hyperexcitability through the blockade of voltage-gated sodium channels. In contrast, neuromodulatory interventions, such as BoNTA, offer a more targeted strategy, without the systemic side effects of oral medications, by directly modulating the release of pain-transmitting neuropeptides and altering the excitability of nociceptive pathways. As such, addressing the underlying pathophysiological mechanisms at both peripheral and central levels, BoNTA represents a significant advancement in the treatment of refractory TN.

## 3. Antinociceptive Mechanisms of BoNTA

BoNTA, a potent neurotoxin produced by the anaerobic bacterium Clostridium botulinum, exerts its classic paralytic effect by cleaving the synaptosomal-associated protein of 25 kDa (SNAP-25). This prevents the exocytosis of acetylcholine at the neuromuscular junction. However, its role in cephalic pain management extends well beyond muscle relaxation [25] as BoNTA exerts its antinociceptive properties by directly antagonizing the pathophysiological processes of TN to eventually inhibit both peripheral and central mechanisms [26,27].

At the periphery, nociceptive sensory neurons, particularly those expressing the transient receptor potential vanilloid 1 (TRPV1) channel, capture BoNTA. The toxin cleaves SNAP-25, effectively blocking the vesicular release of key inflammatory mediators and pain-transmitting neuropeptides, including substance P, CGRP and glutamate, into the synaptic cleft [28,29,30]. Consequently, by inhibiting the release of these substances, BoNTA dampens peripheral sensitization and reduces the neurogenic inflammation that significantly contributes to trigeminal nerve hyperexcitability [31].

Recent studies further demonstrate that BoNTA regulates sensory neuronal channels and modulates voltage-gated calcium channels, thereby reducing neuronal excitability, diminishing calcium influx, and ultimately decreasing neuropeptide exocytosis [32,33].

Beyond inhibiting neuropeptide release at the periphery, emerging data suggest that BoNTA directly affects the expression and function of specific ion channels involved in nociception. In particular, it downregulates the surface expression of TRPV1 channels in sensory neurons [34,35], thereby reducing their sensitivity to noxious thermal and chemical stimuli. Additionally, the toxin may modulate the activity of voltage-gated sodium channels, further reducing the excitability of trigeminal nerve fibers [36]. These peripheral mechanisms jointly reduce nociceptive input to the central nervous system to effectively attenuate the ectopic activity triggering TN paroxysms [37].

Furthermore, evidence demonstrates that BoNTA undergoes retrograde axonal transport from the peripheral injection site to the central nervous system. Studies have identified cleaved SNAP-25 in the trigeminal nucleus caudalis following peripheral administration, providing direct molecular evidence of the toxin’s central translocation [38,39]. This central activity modulates synaptic transmission within the brainstem, further suppressing the release of excitatory neurotransmitters and mitigating central sensitization. Finally, BoNTA also interacts with the endogenous opioidergic and GABAergic systems, enhancing inhibitory pain pathways and promoting a more balanced neurochemical environment within the trigeminal pain matrix [40,41].

The central effects of BoNTA are particularly relevant in refractory TN, where central sensitization plays a prominent role in maintaining chronic pain [42]. By inhibiting the release of glutamate and substance P within the trigeminal nucleus caudalis, the toxin effectively suppresses the excitability of second-order neurons, reducing their responsiveness to incoming nociceptive signals [43]. Its ability to enhance GABAergic and opioidergic transmission synergically acts in achieving pain modulation, reinforcing the descending inhibitory pathways that are often compromised in chronic pain states [44,45]. Noteworthy, the proposed effects of BoNTA on opioidergic and GABAergic neurotransmission should be interpreted with caution as these mechanistic evidences are solely derived mainly from preclinical and non-TN pain models, while direct confirmation of these mechanisms in human TN is currently lacking.

**Figure 1:**
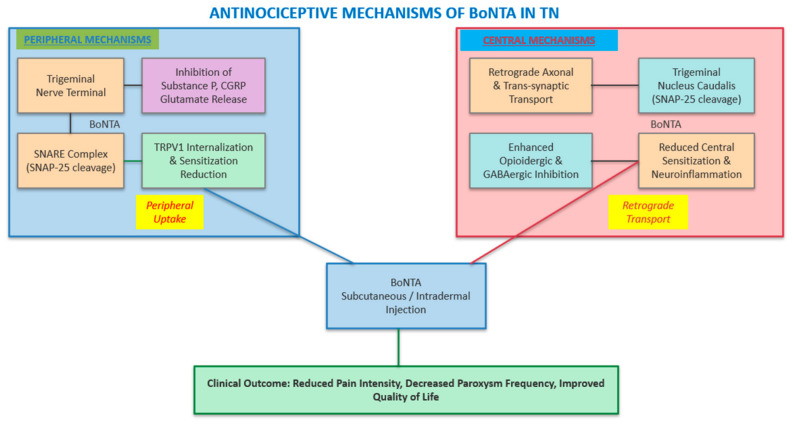
Schematic representation of the peripheral and central antinociceptive mechanisms of BoNTA in TN. Peripheral uptake by TRPV1-expressing nociceptors leads to SNAP-25 cleavage and inhibition of substance P, CGRP, and glutamate release. Retrograde axonal transport delivers the toxin to the trigeminal nucleus caudalis, where it reduces central sensitization and enhances opioidergic and GABAergic inhibition. Author-created schematic based on published mechanistic literature.

Overall, this combined attenuation of peripheral nociceptive input and central hyperexcitability, summarized in Figure 1, provides a pharmacological rationale to support that BoNTA has mechanisms that are biologically plausible and relevant to TN pathophysiology. However, based on these preclinical mechanistic data, we cannot conclude with confidence that BoNTA definitively addresses the underlying pathophysiological mechanisms of TN in humans.

## 4. Clinical Evidence for BoNTA in TN

Data from randomized controlled trials (RCTs), open-label studies and case series support its efficacy and safety profile. The formulations utilized in these studies are onabotulinumtoxinA (Botox^®^ AbbVie Inc. North Chicago, IL 60064, USA) and lanbotulinumtoxinA, both demonstrating comparable therapeutic benefits. A recent comprehensive literature review identified 23 relevant studies and concluded that BoNTA consistently reduces pain intensity and paroxysm frequency in patients with both classical and idiopathic TN, with a favorable safety profile, thereby reinforcing its use in clinical practice [46].

### 4.1. Efficacy Outcomes from Randomized Controlled Trials

A double-blind, placebo-controlled study evaluated the efficacy of intradermal and submucosal injections of 75 units (U) of lanbotulinumtoxinA/Lantox (Lanzhou Biological Products Institute, Sinopharm Plaza, Haidian district, Beijing, China) in 42 patients with classical TN. At the 12-week endpoint, 68.18% of patients in the lanbotulinumtoxinA group achieved a greater than 50% reduction in pain severity, compared to only 15% in the placebo group. The frequency of pain attacks was significantly reduced as early as the first week post-injection, with the therapeutic effect maintained throughout the study period [47].

Subsequent RCTs have replicated these findings. A double-blind, placebo-controlled trial involving 36 patients with classical and idiopathic TN administered 50 U of onabotulinumtoxinA subcutaneously using a “follow the pain” approach. The BoNTA group exhibited a significant reduction in both pain intensity and paroxysm frequency at the two- and three-month follow-ups, whereas the placebo group showed no such improvement. The duration of the antinociceptive effect was at least three months, highlighting the sustained benefit of a single treatment session [48].

In another randomized, double-blind, placebo-controlled trial, 84 patients with classical TN received intradermal/submucosal injections of lanbotulinumtoxinA/Lantox (25 U or 75 U) or placebo. Both active doses produced significant VAS reductions from week 1 through week 8, with response rates of 70.4% (25 U) and 86.2% (75 U) versus 32.1% for placebo. No significant efficacy difference was observed between the two doses. Adverse reactions were mild to moderate, supporting BoNTA as a safe, effective option for refractory trigeminal neuralgia [49].

Finally, in a randomized, single-blind, placebo-controlled trial, 20 Egyptian patients with intractable idiopathic TN received a one-time subcutaneous BoNTA injection (40–60 U), using the “follow the pain” method. At 12 weeks, the BoNTA group demonstrated a mean visual analogue scale (VAS) reduction of 6.5 versus 0.3 for placebo (*p* < 0.0001), alongside significant decreases in acute medication use and meaningful improvements in quality of life [50].

A recent systematic review and meta-analysis evaluated data from 23 studies, including four RCTs and 19 non-RCTs. The pooled analysis revealed that BoNTA significantly decreased mean VAS scores and pain attack frequency from baseline to the end of follow-up. The overall proportion of BoNTA responders was highly significant, with a pooled response rate substantially exceeding that of placebo, further supporting its use as an effective intervention for refractory cases [51]. Another systematic review specifically examined the use of BoNTA in orofacial neuropathic pain conditions, including TN. All selected studies concluded the superiority of BoNTA injections over placebo for reducing pain levels, and five out of six studies highlighted a meaningful improvement in the patient quality of life [52]. Table 1 describes the methodology and results of RCTs testing BoNTA in TN.

### 4.2. Open-Label Studies and Case Series

Beyond RCTs, a growing body of open-label studies and case series further adds to our understanding of BoNTA use in TN. An early open-label study reported reduced pain in all 13 patients with TN treated with BoNTA, with effects lasting for several months, providing early clinical evidence for the antinociceptive potential of this approach [53]. More recently, a case series and literature review from a specialized pain center demonstrated that BoNTA is effective and safe in treating refractory idiopathic TN for up to 12 weeks, with a meaningful reduction in VAS scores and a significant improvement in patient-reported quality of life [54].

A case of successful intraoral injection of BoNTA in an elderly woman with TN who had not responded adequately to subcutaneous injections alone highlighted the potential value of alternative injection routes for specific anatomical distributions of pain [55]. BoNTA exerted a sustained benefit even when a single treatment session is administered as add-on therapy to carbamazepine or oxcarbazepine in 15 treatment-refractory TN patients who failed to respond (less than 30% response rate) to adequate monotherapy [56]. These findings are particularly relevant for clinicians managing patients with TN affecting the V2 and V3 trigeminal branches, where the pain often has a significant orofacial component and can profoundly impair eating [57].

## 5. Dosing Strategies and Injection Techniques

Optimizing dosing and injection protocols is a critical aspect of BoNTA therapy in TN. Despite the availability of clinical data, there remains a lack of universal standardization regarding the optimal dose, dilution and administration route. However, a consensus based on the cumulative experience of specialized pain centers worldwide remains yet an unmet need.

The most widely adopted approach is the “follow the pain” approach. This involves identifying the patient specific pain distribution and trigger zones prior to injecting BoNTA. Clinicians typically accomplish this through a detailed clinical interview and physical examination, supplemented in some centers by quantitative sensory testing [58]. Injections are then administered subcutaneously or intradermally, spaced approximately 1.0 to 1.5 cm apart along the affected trigeminal branches. This targeted delivery ensures that the toxin is concentrated in the areas of maximal nociceptive input, maximizing its peripheral and central effects [59,60].

Dosing regimens vary considerably across studies, ranging from 25 to 100 U per treatment session. As mentioned earlier, a pivotal RCT compared the efficacy of two different doses of lanbotulinumtoxinA (25 U versus 75 U) against a placebo in 84 patients with classical TN. Both the low-dose and high-dose groups demonstrated significant reductions in VAS scores and pain frequency compared to placebo, with no significant difference in efficacy between the two active treatment arms. The response rates at eight weeks were 70.4% for the 25-unit group and 86.2% for the 75-unit group. These findings suggest that lower doses of BoNTA may be sufficient to achieve substantial pain relief, potentially minimizing the risk of dose-dependent adverse effects [49].

In our clinical opinion, a tailored approach is the most reasonable strategy. A starting dose of 25 to 50 U, distributed across the affected dermatomes and trigger zones, might be the reasonable initial step. If the patient experiences inadequate pain relief or a rapid recurrence of symptoms, the dose can be titrated upwards in subsequent sessions, up to a maximum of 100 U, depending on tolerability and clinical response [61,62]. The injection is typically performed under aseptic conditions using a fine-gauge (27 to 30 gauge) and short (8 mm) needle. A practical point is the dose delivered per infiltration site. In published TN studies, per-site dosing generally ranges from 1.25 to 5 U, depending on total dose, dilution (per 0.1 mL sterile normal saline), number of sites, and anatomical region. A starting dose is 2.5–5 U per point should be injected, while lower doses (1.25–2.5 U) should apply near perioral or masticatory muscles to reduce the risk of facial asymmetry or chewing difficulty [40,63]. Nonetheless, considered the protocol heterogeneities, the per-point dose should be individualized according to the extent of the painful territory and trigger-zone mapping and not be viewed as a standard or formally validated guideline.

Table 2 shows an expert-opinion-based, suggested clinical protocol for BoNTA in refractory TN. The 25–75 U dose range is supported by a randomized dose-comparison trial [49], whereas needle gauge, dilution, spacing, and some retreatment decisions are identified as practice-based or expert-opinion-derived parameters, supported by clinical evidence [59,60,61,62,63].

## 6. Long-Term Outcomes and Repeated Injections

While the short-term efficacy of BoNTA is well-documented, the long-term management of refractory TN requires a long-term management plan. The duration of pain relief following a single BoNTA injection typically ranges from three to six months, after which the symptoms gradually relapse [50]. For patients who respond favorably to the initial treatment, repeated injections are often necessary to maintain pain control.

Longitudinal studies demonstrate that the efficacy of BoNTA is generally preserved over multiple treatment cycles [59,64]. A recent retrospective analysis evaluated the outcomes of single versus multiple BoNTA injections in a cohort of TN patients. The study found that while efficacy slightly decreased after multiple rounds of injections, BoNTA remained a highly effective and well-tolerated therapy for patients who chose to continue treatment over an extended period. Notably, patients who experienced a robust response to the first injection were more likely to benefit from subsequent treatments. This highlights the importance of the initial therapeutic trial in predicting long-term success [64].

With accumulated clinical experience, the long-term safety of repeated BoNTA injections has become evident [65]. Unlike systemic pharmacological agents, which often cause cumulative toxicity and dose-limiting side effects, the local administration of BoNTA therapy lowers the risk of systemic complications. As such, there is no evidence, suggesting that repeated BoNTA injections lead to the development of clinically significant neutralizing antibodies or a progressive loss of efficacy over time, making it a good option for the long-term management of refractory TN [64,65]. Furthermore, integrating BoNTA into an individualized pain management plan can significantly improve the patient overall quality of life. It allows them to regain normal daily activities, such as eating, speaking and maintaining oral hygiene, without the constant fear of triggering an attack [56].

Tellingly, while available longitudinal data suggest that the clinical benefit of repeated BoNTA injections is often largely preserved, some cohorts report a modest decrease in efficacy across multiple treatment cycles. Immunogenicity and neutralizing-antibody formation might be the causes for these BoNTA wearing-off phenomena [66]. However, these have not been systematically studied in patients with refractory TN.

## 7. Safety Profile and Adverse Events

The safety and tolerability of BoNTA are critically important, particularly when treating elderly patients with refractory TN who may already be burdened by the side effects of systemic medications. Overall, BoNTA has an excellent safety profile in the context of TN treatment. Adverse events are predominantly mild, localized, and transient [67].

The most frequently reported side effect is facial asymmetry, occurring in approximately 10% to 15% of patients [46]. This asymmetry is a direct consequence of the toxin’s paralytic action on the facial musculature adjacent to the injection sites, particularly when treating the maxillary (V2) and mandibular (V3) branches [68]. While cosmetically noticeable, the asymmetry is typically mild and resolves spontaneously within a few weeks to months as muscle function recovers [69]. Other common, self-limiting adverse events include localized pain, erythema, edema and hematoma at the injection sites [70,71]. These adverse events are generally mild and can be minimized by using fine-gauge needles and applying cold compresses immediately post-procedure [72].

In rare instances, patients may experience transient weakness of the masseter or pterygoid muscles, leading to mild difficulty in chewing or swallowing [46,71]. However, severe or systemic complications, such as generalized weakness or respiratory distress, have not been reported in the context of TN treatment [73]. Absolute contraindications to BoNTA include known hypersensitivity to any botulinum toxin preparation, infection at the proposed injection site and neuromuscular junction disorders, such as myasthenia gravis or Lambert–Eaton syndrome. Relative contraindications include pregnancy, breastfeeding and concurrent use of aminoglycoside antibiotics, which may potentiate the effects of the toxin [74]. Table 3 summarizes the safety profile of BoNTA in TN to represent author synthesized estimates, based on available clinical data. However, the exact percentages should be perceived as approximate clinical ranges, because TN trials reported these events inconsistently and often without pooled event-specific denominators.

Moreover, BoNTA avoids permanent neurological deficits, such as facial numbness and anesthesia dolorosa that can complicate neuroablative surgical procedures [75]. This favorable risk–benefit ratio makes BoNTA an attractive therapeutic option, particularly for patients who are poor surgical candidates or those susceptible to the cognitive burden of high-dose pharmacotherapy.

## 8. Integrating BoNTA into the TN Treatment Algorithm

The management of refractory TN requires a patient-centered approach that carefully considers the benefit–risk ratio of available therapies. Based on the available data, BoNTA should be considered as a significant option in the current TN treatment algorithm, serving as an intermediate step between pharmacological and invasive management.

For patients with classical TN who have failed conventional pharmacotherapy, MVD remains the gold standard, offering the highest probability of long-term, medication-free pain relief [76,77]. Across long term studies, MVD efficacy ranges approximately from 87–98% for immediate/early pain relief. Long-term durability is lower and more phenotype-dependent with about 72% favorable outcome in elderly idiopathic TN—approximately 75% recurrence-free at 96 months in primary TN, but only about 40% recurrence-free in mixed TN [78,79,80]. However, for patients too frail for major surgery, those who decline surgical intervention, or those with idiopathic or secondary TN where MVD is contraindicated, BoNTA may be considered before invasive surgical intervention because it is minimally invasive, repeatable, and generally reversible. Similarly, for patients with TN secondary to multiple sclerosis, where the underlying demyelinating disease precludes surgical decompression, BoNTA offers a valuable non-surgical alternative [81]. However, this sequencing should be individualized according to the specific patient clinical phenotype and even in patients with clear neurovascular compression and acceptable operative risk; BoNTA could be used as an intermediate option before MVD.

On clinical grounds, BoNTA can be utilized as a monotherapy or as add-on treatment [82]. For patients experiencing intolerable side effects from anticonvulsants use, BoNTA may allow a gradual tapering of oral medications, thereby reducing the systemic burden, while maintaining adequate pain control. Alternatively, for patients with partial responses to pharmacotherapy, the addition of BoNTA can provide the necessary synergistic effect to achieve comprehensive pain relief. The combination of low-dose carbamazepine with BoNTA has been reported to be particularly effective in some patients, allowing for a significant reduction in the anticonvulsant dose while maintaining or improving pain control [56].

**Figure 2:**
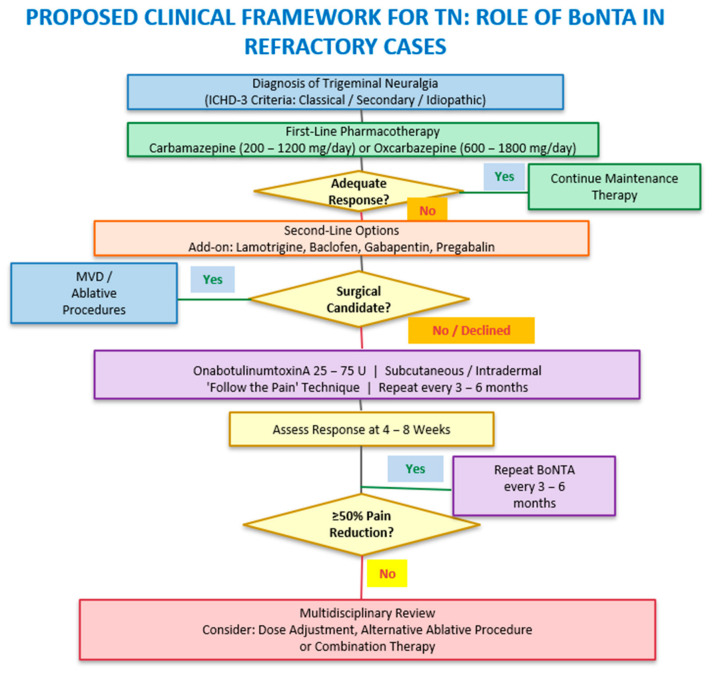
Proposed clinical framework for TN, highlighting the integration of BoNTA in refractory cases. Author-created synthesis based on current diagnostic criteria, treatment recommendations, and published BoNTA clinical literature. MVD: microvascular decompression.

In our opinion, the decision to initiate BoNTA therapy should jointly be made with TN patients, because clinicians must ensure that the patient has realistic expectations regarding the anticipated duration of effect, the need for repeated injection and the potential for transient facial asymmetry. A thorough clinical assessment, including a detailed mapping of the pain distribution and trigger zones, is essential for tailoring the injection protocol to the individual patient’s needs. Patients should be informed that the onset of pain relief typically occurs within 10–15 days after treatment and that the maximum benefit is usually achieved at four to eight weeks [83]. Figure 2 depicts a proposed clinical framework, rather than a definitive guideline, for TN. It also highlights when BoNTA is to be considered, individualized, according to surgical eligibility, patient preference, comorbidity, and tolerance of oral medications. Table 4 describes the pharmacological and invasive treatment option for TN.

## 9. Future Directions for Research

Although the efficacy of BoNTA in refractory TN is well-documented, several questions about its optimal use remain open. First, there is a need for large-scale, multicenter, RCTs with standardized protocols to definitively establish the optimal dose, dilution and injection technique. Such studies would provide the robust data required for regulatory approval, facilitating broader access to this therapy and likely enabling its inclusion in international clinical guidelines [52].

Future research should also focus on identifying clinical and demographic predictors of treatment response. Understanding which patient phenotypes, such as age, disease duration, specific pain characteristics, TN subtype, or the presence of concomitant autonomic symptoms, are most likely to be BoNTA-responsive would help clinicians to provide personalized therapy in order to achieve optimized outcomes [84]. For example, it remains unclear whether patients with classical TN respond differently to BoNTA, compared to those with secondary or idiopathic TN. Similarly, the impact of prior surgical interventions or the presence of comorbid psychological conditions on the efficacy of BoNTA warrants further investigation. Prospective studies incorporating detailed phenotyping, including quantitative sensory testing and advanced neuroimaging, might also provide valuable insights to these issues [85,86].

The exploration of novel BoNTA formulations and delivery methods is also an attractive topic for further study to pursue [87]. The development of longer-acting toxins or targeted delivery systems that minimize diffusion into adjacent musculature could further enhance the efficacy and safety of the treatment, reduce the frequency of injections and mitigate the risk of facial asymmetry [88]. For example, the use of liposomal or nanoparticle-based delivery systems could facilitate the targeted delivery of BoNTA to nociceptive sensory neurons, enhancing its antinociceptive effects, while minimizing off-target side effects [89,90].

Advanced neuroimaging techniques, such as functional (fMRI) and diffusion tensor imaging, could provide valuable insights into the central mechanisms of BoNTA in TN [91]. By elucidating its impact on brainstem connectivity and central sensitization, we can likely refine our understanding of BoNTA’s antinociceptive properties and potentially identify new therapeutic targets within the trigeminal pain pathways. fMRI studies could be used to monitor changes in brain activation patterns following BoNTA treatment, providing objective evidence of its central effects and helping to correlate these changes with clinical outcomes [92]. Similarly, diffusion tensor imaging could be used to assess the structural integrity of the trigeminal nerve and its central projections, providing insights into the long-term effects of BoNTA on neuroplasticity and nerve regeneration [93].

Finally, by identifying specific genetic polymorphisms or circulating biomarkers associated with treatment response, we might be able to develop personalized therapeutic strategies tailored to the individual patient biological profile, significantly improving the efficacy and safety of BoNTA [94].

## 10. Level and Limitations of the Available Evidence

The randomized evidence remains limited in size and duration. Across the four principal placebo-controlled trials, approximately 182 patients were randomized, most studies were single-center, follow-up was generally short, and one trial was single-blind. In the 2024 updated meta-analysis by Hu et al., BoNTA significantly reduced VAS scores in RCTs compared with baseline, but the estimate was accompanied by wide uncertainty (effect size −4.05; 95% CI: −6.13 to −1.97). In non-randomized studies, pooled single-arm analyses showed reductions in VAS scores (effect size −5.19; 95% CI: −6.05 to −4.33) and attack frequency (effect size −17.85; 95% CI: −23.36 to −12.34) [51]. Non-RCT observational findings are limited by heterogeneity, uncontrolled designs, variable outcome definitions, and possible selection and reporting bias. Overall, the available findings should be interpreted cautiously because of the above-described limitations.

## 11. Conclusions

BoNTA is a minimally invasive and generally well-tolerated therapeutic option for patients with refractory who have failed or are intolerant of conventional pharmacological therapies or those not eligible for surgical approaches TN. By modulating both peripheral nociceptive input and central hyperexcitability, BoNTA addresses the complex pathophysiology of TN at multiple levels, targeting the mechanisms that characterize the refractory phenotype. Current evidence suggests meaningful reductions in pain intensity and attack frequency, with mainly transient local adverse effects. However, the randomized evidence remains limited, with short follow-up and heterogeneous injection protocols. Larger multicenter trials using standardized dosing, injection techniques, and long-term safety assessment are needed to better define its role in refractory TN management.

## Figures and Tables

**Table 1:** Summary of key randomized controlled trials of BoNTA in TN.

Study	Study Design	N	Intervention	Key Efficacy Outcomes
**Wu et al. (2012)** [47]	Double-blind, placebo-controlled	42	LanbotulinumtoxinA 75 U vs. Placebo	68.18% responders (>50% pain reduction) vs. 15% placebo at 12 weeks. Significant reduction in attack frequency from week 1.
**Zúñiga et al. (2013)** [48]	Double-blind, placebo-controlled	36	OnabotulinumtoxinA 50 U vs. Placebo	Significant reduction in VAS and paroxysm frequency at 2 and 3 months. No significant change in placebo group.
**Zhang et al. (2014)** [49]	Double-blind, placebo-controlled	84	LanbotulinumtoxinA 25 U vs. 75 U vs. Placebo	70.4% (25 U) and 86.2% (75 U) responders at 8 weeks vs. 32.1% placebo. No significant difference between active doses.
**Shehata et al. (2013)** [50]	Double-blind, placebo-controlled	20	OnabotulinumtoxinA 40–60 U vs. Placebo	Mean VAS reduction 6.5 at 12 weeks vs. 0.3 placebo. Significant reduction in paroxysm frequency.

**Table 2:** Recommended clinical protocol for BoNTA administration in refractory TN.

Parameter	Recommendation
**Formulation**	OnabotulinumtoxinA or LanbotulinumtoxinA
**Initial dose**	25–50 U
**Maximum dose**	75–100 U (titrated based on response and tolerability)
**Dilution**	0.9% sterile saline (1.25–5.0 U per 0.1 mL)
**Dose per infiltration point**	Usually 1.25–5 U per point; commonly 2.5–5 U per point; consider 1.25–2.5 U per point in regions at higher risk of diffusion-related weakness or facial asymmetry
**Injection technique**	“Follow the pain” targeting trigger zones and affected dermatomes
**Route**	Subcutaneous or Intradermal
**Needle gauge**	27–30 gauge
**Spacing**	1.0 to 1.5 cm between injection sites
**Re-treatment interval**	Every 3 to 6 months, depending on symptom recurrence
**Pre-procedure assessment**	Detailed pain mapping; quantitative sensory testing where available

**Table 3:** Adverse events associated with BoNTA in TN.

Adverse Event	Estimated Incidence	Characteristics and Management
**Facial asymmetry** [46,68]	10–15%	Transient, mild to moderate. Resolves spontaneously within weeks to months. More common with V2/V3 injections.
**Injection site pain** [70]	5–10%	Mild, self-limiting. Managed with ice packs post-injection.
**Edema/erythema** [71]	5–8%	Localized inflammatory response. Resolves within days.
**Hematoma** [70]	<5%	Minor bruising at injection sites. Minimized by avoiding intravascular injection.
**Masticatory weakness** [46,71]	Rare (<2%)	Transient difficulty chewing. Associated with higher doses or deep injections near masseter/pterygoid muscles.
**Systemic complications** [73]	Not reported	No cases of generalized weakness or respiratory distress in TN treatment context.

**Table 4:** Comparison of therapeutic options for refractory TN.

Treatment	Mechanism	Efficacy	Side Effects	Suitable Phenotype
**Carbamazepine/Oxcarbazepine**	Sodium channel blockade	High (short-term)	Cognitive impairment, ataxia, hyponatraemia, hepatotoxicity	First-line; all TN subtypes
**Microvascular Decompression**	Surgical decompression of trigeminal root	Very high (long-term)	Posterior fossa complications, CSF leak, cranial nerve palsy	Classical TN; good surgical candidates
**Gamma Knife Radiosurgery**	Ablation of trigeminal root	Moderate-high	Facial numbness, delayed effect	Elderly; poor surgical candidates
**Radiofrequency Thermocoagulation**	Ablation of Gasserian ganglion	Moderate-high	Facial numbness, anesthesia dolorosa	Elderly; poor surgical candidates
**BoNTA**	Peripheral and central neuromodulation	Moderate-high	Transient facial asymmetry, injection site reactions	Refractory TN; poor surgical candidates; adjunct to pharmacotherapy

## Data Availability

No new data were created or analyzed in this study.

## Abbreviations

The following abbreviations are used in this manuscript:

TN	Trigeminal neuralgia
MVD	Microvascular decompression
BoNTA	Botulinumtoxin type-A
CGRP	Calcitonin gene-related peptide
SNAP-25	Synaptosomal-associated protein of 25 kDa
TRPV1	Transient receptor potential vanilloid 1
RCTs	Randomized controlled trials
Botox^®^	OnabotulinumtoxinA
U	Units
